# Vascular depression – regarding a case report

**DOI:** 10.1192/j.eurpsy.2021.1146

**Published:** 2021-08-13

**Authors:** P. Felgueiras, A. Martins, A. Miguel, N. Almeida

**Affiliations:** Psychiatry, Vila Nova de Gaia Hospital Center, Vila Nova de Gaia, Portugal

**Keywords:** Vascular Depression, Geriatric depressive syndromes, Cardiovascular disease, Subcortical white matter abnormalities

## Abstract

**Introduction:**

Age-related vascular changes have long been documented as an etiopathogenic factor of some geriatric depressive syndromes. More recently, it has emerged the concept of “Vascular Depression” recognizing that cardiovascular disease may predispose, precipitate or perpetuate late life depression. This condition was defined by an episode of major depressive disorder within the preceding 12 months in elderly with cardiovascular/cerebrovascular disease, or major cardiovascular risk factors. Vascular Depression isn`t described in DSM-V, and that difficults clinical recognition and affects clinically informed systematic studies.

**Objectives:**

Regarding a clinical case, we enphasize the clinical impact of Vascular Depression`s hypothesis.

**Methods:**

We present a qualitative review of this topic using the Pubmed Central database.

**Results:**

74 years old male patient, with major depressive disorder about ten years. Depressive and cognitive symptoms didn`t respond to antidepressive treatment and his functional state has gradually declined.

**Conclusions:**

Vascular depression develops after the 60 – 65 years in the absence of personal and family history of affective disorder. The key symptoms are low energy, anhedonia, deficits in selfinitiation, psychomotor retardation, reduced processing speed and lack of insight into mood symptoms. Clinical assessment includes a review of history of vascular risk factors or/and disease, but also an imagiological evidence demonstrating subcortical white matter abnormalities. Insidious and chronic course tends to delay its recognition and management. This becomes critical because Vascular Depression is associated with poor response to antidepressant treatment and persistent depressive symptoms. It`s also associated with poor selfmanagement of comorbidities and impairment in daily function. Increased mortality from all causes is widely documented.

